# Supplementary material for: Essential genes encoded by the mating-type locus of the human fungal pathogen Cryptococcus neoformans
Source: mBio. 2025 Feb 25;16(4):e00223-25. doi: 10.1128/mbio.00223-25 (PMC11980393; doi:10.1128/mbio.00223-25)
Supplement: Supplemental material — Tables S1 to S5; Fig. S1 to S6. [file mbio.00223-25-s0001.docx]

**SUPPLEMENTAL TABLES**

**Table S1. Strains used in this study.**

| **Strain** | **Background strain** | **Genotype** | **Note** |
| --- | --- | --- | --- |
| H99α |  | WT | (1) |
| KN99**a** |  | WT | (1) |
| KN99α |  | WT | (1) |
| CnLC6683 |  | WT | (2) |
| ZB225 | CnLC6683 | *myo2***a**Δ::*NAT*/*MYO2*α | This study |
| ZB505 | CnLC6683 | *myo2***a**Δ::*NAT*/*MYO2*α | This study |
| ZB226 | CnLC6683 | *prt1***a**Δ::*NAT* /*PRT1*α | This study |
| ZB228 | CnLC6683 | *prt1***a**Δ::*NAT* /*PRT1*α | This study |
| ZB229 | CnLC6683 | *rpl22***a**Δ::*NAT* /*RPL22*α | This study |
| ZB507 | CnLC6683 | *rpl22***a**Δ::*NAT* /*RPL22*α | This study |
| ZB231 | CnLC6683 | *rpl39***a**Δ::*NAT* /*RPL39*α | This study |
| ZB511 | CnLC6683 | *rpl39***a**Δ::*NAT* /*RPL39*α | This study |
| ZB314 | CnLC6683 | *rpo41***a**Δ::*NAT* /*RPO41*α | This study |
| ZB315 | CnLC6683 | *rpo41***a**Δ::*NAT* /*RPO41*α | This study |
| ZB237 | CnLC6683 | *MYO2***a/***myo2*αΔ::*NEO* | This study |
| ZB318 | CnLC6683 | *PRT1***a/***prt1*αΔ::*NEO* | This study |
| ZB512 | CnLC6683 | *PRT1***a/***prt1*αΔ::*NEO* | This study |
| ZB238 | CnLC6683 | *RPL22***a/***rpl22*αΔ::*NEO* | This study |
| ZB583 | CnLC6683 | *RPL22***a/***rpl22*αΔ::*NEO* | This study |
| ZB232 | CnLC6683 | *RPL39***a/***rpl39*αΔ::*NEO* | This study |
| ZB509 | CnLC6683 | *RPL39***a/***rpl39*αΔ::*NEO* | This study |
| ZB234 | CnLC6683 | *RPO41***a/***rpo41*αΔ::*NEO* | This study |
| ZB241 | CnLC6683 | *RPO41***a/***rpo41*αΔ::*NEO* | This study |
| ZB478 | KN99**a** | P_tet_-*MYO2***a** | This study |
| ZB479 | KN99**a** | P_tet_-*MYO2***a** | This study |
| ZB478 | H99α | P_tet_-*MYO2*α | This study |
| ZB479 | H99α | P_tet_-*MYO2*α | This study |
| ZB669 | H99α | P_2x_*_CTR4_*-*TOR1* | (3) |
| ZB670 | KN99**a** | P_2x_*_CTR4_*-*PRT1***a** | This study |
| ZB671 | KN99**a** | P_2x_*_CTR4_*-*PRT1***a** | This study |
| ZB235 | KN99**a** | *myo2***a**Δ::*NAT* | This study |
| ZB236 | KN99**a** | *myo2***a**Δ::*NAT* | This study |
| ZB242 | KN99α | *myo2*αΔ::*NEO* | This study |
| ZB243 | KN99α | *myo2*αΔ::*NEO* | This study |
| ZB487 | KN99**a**, recombinant mitochondria | Hem15-GFP--*NAT* Nop1-mCherry--*NEO* | This study |
| ZB307 | KN99α, recombinant mitochondria | *myo2*Δ::*NEO* | This study |
| ZB308 | KN99α, recombinant mitochondria | *myo2*Δ::*NEO* | This study |

**Table S2. Primers used in this study.**

| **Name** | **Squence 5'-3'** | **Purpose** |
| --- | --- | --- |
| JOHE45803/SS | CTCCACCCTTACTGATCGCC | *MYO2*_**a**_Upstream_Forward |
| JOHE45804/SS | GTCATAGCTGTTTCCTGAGTACCAGGAACCCAGCTAGT | *MYO2*_**a**_Upstream_Reverse_M13 |
| JOHE45805/SS | CTGGCCGTCGTTTTACTATGCGTGCAGATGTACCGG | *MYO2*_**a**_Downstream_Forward_M13 |
| JOHE45806/SS | ACTCCTTCCTCGGCTCTTCT | *MYO2*_**a**_Downstream_Reverse |
| JOHE45807/SS | CGAGAATGAAGAGGGCTGGG | *PRT1*_**a**_Upstream_Forward |
| JOHE45808/SS | GTCATAGCTGTTTCCTGCACTGACTTTTCCTCGATGTCC | *PRT1*_**a**_Upstream_Reverse_M13 |
| JOHE45809/SS | CTGGCCGTCGTTTTACCAGAGAGATCGGGGAGTGGA | *PRT1*_**a**_Downstream_Forward_M13 |
| JOHE45810/SS | ACACAATCAATGCGCCTGC | *PRT1*_**a**_Downstream_Reverse |
| JOHE45811/SS | ACCTTCACCTCAAGCTCTGC | *RPL22*_**a**_Upstream_Forward |
| JOHE45812/SS | GTCATAGCTGTTTCCTGGCAGCATCCACAACCCAAAA | *RPL22*_**a**_Upstream_Reverse_M13 |
| JOHE45813/SS | CTGGCCGTCGTTTTACGTGTCGTTGCCACTTCCAAG | *RPL22*_**a**_Downstream_Forward_M13 |
| JOHE45814/SS | TGATGTCGGCAACCTTCCTC | *RPL22*_**a**_Downstream_Reverse |
| JOHE45815/SS | TTTCCATCCTCGTCGCTGTT | *RPL39*_**a**_Upstream_Forward |
| JOHE45816/SS | GTCATAGCTGTTTCCTGGCGTCGAACCAAGCTTAACC | *RPL39*_**a**_Upstream_Reverse_M13 |
| JOHE45817/SS | CTGGCCGTCGTTTTACCAAACAAAGTCTGCCGTGGG | *RPL39*_**a**_Downstream_Forward_M13 |
| JOHE45818/SS | CACTGCATCAATCGAACGCC | *RPL39*_**a**_Downstream_Reverse |
| JOHE45819/SS | GCGCACAACACTCCATTTAGAC | *RPO41*_**a**_Upstream_Forward |
| JOHE45820/SS | GTCATAGCTGTTTCCTGGATGTCTTGCCGCCTTGTCC | *RPO41*_**a**_Upstream_Reverse_M13 |
| JOHE45821/SS | CTGGCCGTCGTTTTACCCTCTCCCCACTGCTCTACT | *RPO41*_**a**_Downstream_Forward_M13 |
| JOHE45822/SS | GCTTGGGTCTACTATGTCTGTCG | *RPO41*_**a**_Downstream_Reverse |
| JOHE45823/SS | TTCTGAAGTCCCTCGCAACC | *MYO2*_alpha_Upstream_Forward |
| JOHE45824/SS | GTCATAGCTGTTTCCTGGGATCTGTTGTCGCGCTTTG | *MYO2*_alpha_Upstream_Reverse_M13 |
| JOHE45825/SS | CTGGCCGTCGTTTTACCAGACGATGTGGGCCCATAT | *MYO2*_alpha_Downstream_Forward_M13 |
| JOHE45826/SS | TATCTCAGCAGCCACGCAAA | *MYO2*_alpha_Downstream_Reverse |
| JOHE45827/SS | TCGGACACCTCAGCTGTAGA | *PRT1*_alpha_Upstream_Forward |
| JOHE45828/SS | GTCATAGCTGTTTCCTGGCGAGAACAGAGGCAAGTCA | *PRT1*_alpha_Upstream_Reverse_M13 |
| JOHE45829/SS | CTGGCCGTCGTTTTACGCTCAGCTTCAATCTCACGC | *PRT1*_alpha_Downstream_Forward_M13 |
| JOHE45830/SS | GGAACCAGTCATCCCCACTC | *PRT1*_alpha_Downstream_Reverse |
| JOHE45831/SS | CCGGCGTGATGGTAGTGATA | *RPL22*_alpha_Upstream_Forward |
| JOHE45832/SS | GTCATAGCTGTTTCCTGGTAGTGGAGGGAGCTTTCGG | *RPL22*_alpha_Upstream_Reverse_M13 |
| JOHE45833/SS | CTGGCCGTCGTTTTACTTGCCACTTCCAAGGACACC | *RPL22*_alpha_Downstream_Forward_M13 |
| JOHE45834/SS | CCACTTCATCGACGGTAGGG | *RPL22*_alpha_Downstream_Reverse |
| JOHE45847/SS | CGGTATTTCTGAACGCTCCG | *RPL39*_alpha_Upstream_Forward_4 |
| JOHE45848/SS | GTCATAGCTGTTTCCTGGTCGTAGGAGAATAAGGCGGA | *RPL39*_alpha_Upstream_Reverse_4_M13 |
| JOHE45837/SS | CTGGCCGTCGTTTTACTTCCGCCTGAAGACTGACAG | *RPL39*_alpha_Downstream_Forward_M13 |
| JOHE45838/SS | TCGGCCTTGAATGATGACCA | *RPL39*_alpha_Downstream_Reverse |
| JOHE45839/SS | GCTGGGAGTGTCAAAAGCGA | *RPO41*_alpha_Upstream_Forward |
| JOHE45840/SS | GTCATAGCTGTTTCCTGGCAGGAAAGCAGAATTGGGTC | *RPO41*_alpha_Upstream_Reverse_M13 |
| JOHE45841/SS | CTGGCCGTCGTTTTACCCTCTCCCCACTGCTCTACT | *RPO41*_alpha_Downstream_Forward_M13 |
| JOHE45842/SS | TGGGTCTACTATGTCTGTCGA | *RPO41*_alpha_Downstream_Reverse |
| JOHE45853/SS | ATCGATTGGGCCTTCATCTC | *MYO2*_**a**_Internal_Forward |
| JOHE45854/SS | AGGAATCCATGGCCGCATTG | *MYO2*_**a**_Internal_Reverse |
| JOHE45855/SS | AATCAGCTGGAAATTCATTG | *MYO2*_alpha_Internal_Forward |
| JOHE45856/SS | CTGGGATGGAGCTTCTGG | *MYO2*_alpha_Internal_Reverse |
| JOHE45857/SS | CCAATCCAGAAAGAGATGGC | *PRT1*_**a**_Internal_Forward |
| JOHE45858/SS | GGTCCAACGCCATTACATTAG | *PRT1*_**a**_Internal_Reverse |
| JOHE45859/SS | AATCATCAATCTAGTTGCCA | *PRT1*_alpha_Internal_Forward |
| JOHE45860/SS | TTGATATCATGCCAATAATGAC | *PRT1*_alpha_Internal_Reverse |
| JOHE45861/SS | TCGCCGCTTTTGAGAAGTTT | *RPL22*_**a**_Internal_Forward |
| JOHE45862/SS | TCTTGGTAAGGTACTTAAGA | *RPL22*_**a**_Internal_Reverse |
| JOHE45863/SS | TTGCCGCGTTTGAGAAGTTC | *RPL22*_alpha_Internal_Forward |
| JOHE45864/SS | TCTTCGTAAGGTACTTGAGG | *RPL22*_alpha_Internal_Reverse |
| JOHE45865/SS | TCACGATATCGACAACACCGC | *RPO41*_**a**_Internal_Forward |
| JOHE45866/SS | TTTGACGTTGTGCTCGAGTC | *RPO41*_**a**_Internal_Reverse |
| JOHE45867/SS | TCACGATATCAACGACACCGG | *RPO41*_alpha_Internal_Forward |
| JOHE45866/SS | TTTGACGTTGTGCTCGAGTC | *RPO41*_alpha_Internal_Reverse |
| JOHE45868/SS | GGTTAAGCTTGGTTCGACGCCA | *RPL39*_**a**_Internal_Forward |
| JOHE45869/SS | ATGCTGACTCCTGTCCCAAATT | *RPL39*_**a**_Internal_Reverse |
| JOHE45870/SS | GGTTGAGCTTGGTTCGACGCCA | *RPL39*_alpha_Internal_Forward |
| JOHE45871/SS | ATGCTGACGTCTGCCCTCAATC | *RPL39*_alpha_Internal_Reverse |
| JOHE45876/SS | AGATCGATGGTGGGGAGGAA | *MYO2*_**a**_Junction_Forward |
| JOHE45877/SS | CCAAGATGTTTACGTTCGGG | *MYO2*_**a**_Junction_Reverse |
| JOHE45878/SS | GGAACCAGTCATCCCCACTC | *MYO2*_alpha_Junction_Forward |
| JOHE45879/SS | GCAAAGGACCCATCTCAGCT | *MYO2*_alpha_Junction_Reverse |
| JOHE45880/SS | CGAGACGACTGGAATGGTGT | *PRT1*_**a**_Junction_Forward |
| JOHE45881/SS | AGCCATGTGTGAATCCTGCG | *PRT1*_**a**_Junction_Reverse |
| JOHE45884/SS | AGAACTGTGCCCGGAATAGC | *PRT1*_alpha_Junction_Forward2 |
| JOHE45885/SS | AGGGCTTGCCGAAGAACAAT | *PRT1*_alpha_Junction_Reverse2 |
| JOHE45888/SS | GACCAATGCCGGAAGAGGAT | *RPL22*_**a**_Junction_Forward |
| JOHE45889/SS | GTGAGTACCGCATTACCAGC | *RPL22*_**a**_Junction_Reverse |
| JOHE45890/SS | CTTCAGCCTCAGACTCACCC | *RPL22*_alpha_Junction_Forward |
| JOHE45891/SS | CCATTGTCCATGTTCCCCGT | *RPL22*_alpha_Junction_Reverse |
| JOHE45892/SS | CCAAGTCTCTGCTTCCACCA | *RPL39*_**a**_Junction_Forward |
| JOHE45893/SS | TGAGGACAGATTGGCGTGAG | *RPL39*_**a**_Junction_Reverse |
| JOHE45894/SS | CCCTTTCCATCACCTCCGAT | *RPL39*_alpha_Junction_Forward |
| JOHE45895/SS | GGGTTGAAGCTGGGGAGAAC | *RPL39*_alpha_Junction_Reverse |
| JOHE45896/SS | GGTTCGGGCGCTAAGTAACA | *RPO41*_**a**_Junction_Forward |
| JOHE45897/SS | ATCTCGCCGCAAATACCACT | *RPO41*_**a**_Junction_Reverse |
| JOHE45898/SS | TTTAGGCATGGACGCACAGT | *RPO41*_alpha_Junction_Forward |
| JOHE45899/SS | ATCTCGCCGCAAATACCACT | *RPO41*_alpha_Junction_Reverse |
| JOHE52755ZB26 | ACCGGCAGGGTATACTGTTGGCGCTTTGTAAGGTGGACAAGTTTTAGAGCTAGAAATAGC | *MYO2*_alpha_5'_gRNA |
| JOHE52756ZB27 | ACCGGCAGGGTATACTGTTGGCTTGGTAGACGTCCCAGCGGTTTTAGAGCTAGAAATAGC | *MYO2*_alpha_3'_gRNA |
| JOHE52757ZB28 | ACCGGCAGGGTATACTGTTGATGTCCGCCCCCTACAGAAAGTTTTAGAGCTAGAAATAGC | *MYO2*_**a**_5'_gRNA |
| JOHE52758ZB29 | ACCGGCAGGGTATACTGTTGCTGCCAATCGCCGGACAGATGTTTTAGAGCTAGAAATAGC | *MYO2*_**a**_3'_gRNA |
| JOHE52890/ZB109 | ACCGGCAGGGTATACTGTTGTATGCTGTGACCGCTCAGAAGTTTTAGAGCTAGAAATAGC | *PRT1*_**a**_5'_gRNA |
| JOHE52891/ZB110 | ACCGGCAGGGTATACTGTTGCTGGGCACTGGGGAACATTTGTTTTAGAGCTAGAAATAGC | *PRT1*_**a**_3'_gRNA |
| JOHE52892/ZB111 | ACCGGCAGGGTATACTGTTGGCAGCAGCTGGTACTAACAAGTTTTAGAGCTAGAAATAGC | *PRT1*_alpha_5'_gRNA |
| JOHE52893/ZB112 | ACCGGCAGGGTATACTGTTGTCCTCCAACGCTGCTTAGTAGTTTTAGAGCTAGAAATAGC | *PRT1*_alpha_3'_gRNA |
| JOHE52760ZB31 | ACCGGCAGGGTATACTGTTGCAAGTACTTCAAGGTTGATCGTTTTAGAGCTAGAAATAGC | *RPL22*_alpha_3'_gRNA_GI |
| JOHE52761ZB32 | ACCGGCAGGGTATACTGTTGGTTTGCAATCAGGATTACATGTTTTAGAGCTAGAAATAGC | *RPL22*_alpha_5'_gRNA |
| JOHE52763ZB34 | ACCGGCAGGGTATACTGTTGAGTTGCAATGAGGGTTACATGTTTTAGAGCTAGAAATAGC | *RPL22*_**a**_5'_gRNA_GI |
| JOHE52765ZB36 | ACCGGCAGGGTATACTGTTGTGTATCTACACGTTACTAACGTTTTAGAGCTAGAAATAGC | *RPL22*_**a**_gRNA3_GI |
| JOHE52894/ZB113 | ACCGGCAGGGTATACTGTTGCCACGGCAGACTTTGTTTGCGTTTTAGAGCTAGAAATAGC | *RPL39*_**a**_5'_gRNA |
| JOHE52895/ZB114 | ACCGGCAGGGTATACTGTTGATAACGCCAAGCGTCGTCATGTTTTAGAGCTAGAAATAGC | *RPL39*_**a**_3'_gRNA |
| JOHE52896/ZB115 | ACCGGCAGGGTATACTGTTGGGAGTCGTAGGAGAATAAGGGTTTTAGAGCTAGAAATAGC | *RPL39*_alpha_5'_gRNA |
| JOHE52897/ZB116 | ACCGGCAGGGTATACTGTTGAGGCGGAACCACTGAGGAAGGTTTTAGAGCTAGAAATAGC | *RPL39*_alpha_3'_gRNA |
| JOHE52898/ZB117 | ACCGGCAGGGTATACTGTTGGAGCACTAGACCCATGGTTGGTTTTAGAGCTAGAAATAGC | *RPO41*_**a**_5'_gRNA |
| JOHE52899/ZB118 | ACCGGCAGGGTATACTGTTGGAGGATTTCCTTGACCGGTAGTTTTAGAGCTAGAAATAGC | *RPO41*_**a**_3'_gRNA |
| JOHE52900/ZB119 | ACCGGCAGGGTATACTGTTGGTGACAGCGGCTTTGGATTCGTTTTAGAGCTAGAAATAGC | *RPO41*_alpha_5'_gRNA |
| JOHE52901/ZB120 | ACCGGCAGGGTATACTGTTGTTTCTACGATCTTATCGGCAGTTTTAGAGCTAGAAATAGC | *RPO41*_alpha_3'_gRNA |
| JOHE52738ZB9 | TGTAAAACGACGGCCAGT | M13F |
| JOHE52739ZB10 | CAGGAAACAGCTATGAC | M13R |
| JOHE52740ZB11 | CATGCATCTAGGTCTAGAAACC | Cas9-F |
| JOHE52741ZB12 | CCTCTTCACGTGGACGCTCC | Cas9-R |
| JOHE52742ZB13 | GCCCTAGTCCATTGCGAACG | U6-F |
| JOHE52743ZB14 | CAACAGTATACCCTGCCGGTG | U6-R |
| JOHE52744ZB15 | GTTTTAGAGCTAGAAATAGCAAG | sgRNA-F |
| JOHE52745ZB16 | AAGATACTCGATTTGCCGTCC | sgRNA-R |
| JOHE52746ZB17 | GCTCATGGATCCTTTGCATTAGAACTAAAAACAAAGCA | sgRNA-final-F |
| JOHE52747ZB18 | GATCATCCGCGGTAAAACAAAAAAGCACCGACTCGGTGCC | sgRNA-final-R |
| JOHE54481/YC183 | TAGGCCCCTTTTCCGTCTAT | *PRT1***a** *CTR4* promoter replacement L1 |
| JOHE54482/YC184 | CACTCGAATCCTGCATGCTTTTGCGTATGGCGTGGTG | *PRT1***a** *CTR4* promoter replacement L2 |
| JOHE54483/YC185 | CGACAACGACTTCACCAATCATGTCGGTCACCGACTTAAC | *PRT1***a** *CTR4* promoter replacement R1 |
| JOHE54484/YC186 | GCCATCTCTTTCTGGATTGG | *PRT1***a** *CTR4* promoter replacement R2 |
| JOHE54314/ZB363 | GCATGCAGGATTCGAGTG | NAT/CTR-L |
| JOHE54315/ZB364 | GATTGGTGAAGTCGTTGTCG | NAT/CTR-R |
| JOHE54314/ZB366 | AAGGTGTTCCCCGACGACGAATCG | NAT-SM1 |
| JOHE54315/ZB367 | CGATTCGTCGTCGGGGAACACCTT | NAT-SM2 |
| JOHE53219/ZB245 | ACCGGCAGGGTATACTGTTGCTGACTTAGTTAGCTAAGATGTTTTAGAGCTAGAAATAGC | *PRT1***a**_dox_gRNA_1 |
| JOHE53194/ZB220 | ATCGCTGTATGTCTCCGAATCG | *PRT1***a**_dox_internal-R |
| JOHE53195/ZB221 | ATACTTGTTGAAACGGGCTATG | *PRT1***a**_dox_internal-F |
| JOHE54719/YC99 | TGTGGATGCTGGCGGAGGATA | B79 5' screening primer, Screening oligo on *ACT* promoter |
| JOHE54719/YC100 | TTCCCACCCTCAGCAACGCC | J12579 3' screening primer, Screening oligo on *TRP* terminator |
| JOHE53175/ZB201 | TAACAACGGGGTCCAGAAATCG | *MYO2***a**_dox_Upstream-F |
| JOHE53176/ZB202 | CACTGGCCGTCGTTTTACAAGAGTATGTGAGATGAGTGATGC | *MYO2***a**_dox_Upstream-R |
| JOHE53177/ZB203 | CATGGTCATAGCTGTTTCCTATGTCCGCCCCCTACAGAAAA | *MYO2***a**_dox_Downstream-F |
| JOHE53178/ZB204 | AGGAGTAAACGGATTAACCGAC | *MYO2***a**_dox_Downstream-R |
| JOHE53179/ZB205 | CGTCTTTACGTCTCATTATTGG | *MYO2***a**_dox_Internal-F |
| JOHE53180/ZB206 | GTACTGGTAGCGAACCAATTT | *MYO2***a**_dox_Internal-R |
| JOHE53181/ZB207 | CGTCGGGAGGTCGATTTTTCTA | *MYO2***a**_dox_Junction-F |
| JOHE53182/ZB208 | CCCTTCTTCTGACCCGAGTATA | *MYO2***a**_dox_Junction-R |
| JOHE53183/ZB209 | AGCTGTGTTCCTTACGCTGCAA | *MYO2*α_dox_Upstream-F |
| JOHE53184/ZB210 | CACTGGCCGTCGTTTTACAACAGGCCCTTGTGACAAGATTCG | *MYO2*α_dox_Upstream-R |
| JOHE53185/ZB211 | CATGGTCATAGCTGTTTCCTATGACTTCTTTATATTCAAAGG | *MYO2*α_dox_Downstream-F |
| JOHE53186/ZB212 | GTTGAGTGGAGTAAACGGATTC | *MYO2*α_dox_Downstream-R |
| JOHE53187/ZB213 | ATACCTTATAGACTCAGTAGCG | *MYO2*α_dox_Internal-F |
| JOHE53188/ZB214 | ACGAGGACGTCTAATGGGTTG | *MYO2*α_dox_Internal-R |
| JOHE53189/ZB215 | CATGACACTGATGTACATCGCC | *MYO2*α_dox_Junction-F |
| JOHE53190/ZB216 | CCTTCTTTTGACCTGCGTACAG | *MYO2*α_dox_Junction-R |
| JOHE53215/ZB241 | ACCGGCAGGGTATACTGTTGGAACAGTCTTCTGAAAATTGGTTTTAGAGCTAGAAATAGC | *MYO2***a**_dox_gRNA |
| JOHE53217/ZB243 | ACCGGCAGGGTATACTGTTGTCTAAATATCATCCGGCAAGGTTTTAGAGCTAGAAATAGC | *MYO2*α_dox_gRNA |

**Table S3. Quantification of morphology phenotype of** ***myo2*****aΔ and** ***myo2*α****Δ**

|  | Single cells (%) | Clusters with two cells (%) | Clusters with >2 cells (%) |
| --- | --- | --- | --- |
| H99 | 71 ± 9^A^ | 29 ± 9^A^ | 0 ± 0^A^ |
| *myo2***a**Δ | 11 ± 5^B^ | 60 ± 6^B^ | 29 ± 2^B^ |
| *myo2*αΔ | 24 ± 9 ^B^ | 50 ± 10^B^ | 27 ± 2^B^ |

Values are presented as mean ± SE that have been calculated based on three independent experiments. At least 100 cells or clusters were examined for each strain. For the values in the same column, those with different superscript letters (i.e. xx^A^ or xx^B^) are significantly different statistically (one-way ANOVA with Dunnett’s multiple comparisons test, P < 0.05).

**Table S4.** **Quantification of cytokinesis phenotype of *myo2*aΔ and *myo2*αΔ**

|  | Small bud neck without chitin accumulation (%) | Wide bud neck with accumulated chitin (%) |
| --- | --- | --- |
| H99 | 97 ± 6^A^ | 3 ± 6^A^ |
| *myo2***a**Δ | 16 ± 13^B^ | 84 ± 16^B^ |
| *myo2*αΔ | 17 ± 17^B^ | 83 ± 17^B^ |

For comparison, only two-cell clusters resembling budding were included in the analysis. Values are presented as mean ± SE that have been calculated based on three independent experiments, and a minimum of 30 clusters were examined for each strain. For the values in the same column, those with different superscript letters (i.e. xx^A^ or xx^B^) are significantly different statistically (one-way ANOVA with Dunnett’s multiple comparisons test, P < 0.05).

**Table S5. Mapped reads for Ribo-seq and RNA-seq experiments.**

| **Repeat #** | **Ribo-seq combined sample number for sequencing** | **Sample Description** | **Ribo-seq mapped reads** | **RNA-seq mapped reads** |
| --- | --- | --- | --- | --- |
| 1 | 2-129-1 | KN99**a** X H99α (F) | 1802289 | 17017502 |
|  |  | YFF96α (*rpl22*α::*RPL22***a**) x KN99**a** | 1248887 | 19812632 |
|  |  | YFF113**a** (*rpl22***a**::*RPL22*α) x H99α (F) | 406038 | 19680437 |
|  |  | YFF96α (*rpl22*α::*RPL22***a**) x YFF113**a** (*rpl22***a**::*RPL22*α) | 800679 | 14543343 |
|  |  | YFF113**a** (*rpl22***a**::*RPL22*α) | 812059 | 19536864 |
|  |  | YFF96α (*rpl22*α::*RPL22***a**) | 1701071 | 20006780 |
|  |  | KN99**a** | 1066209 | 18421800 |
|  |  | H99α (F) | 1401750 | 12527323 |
| 2 | 2-129-2 | KN99**a** X H99α (F) | 328441 | 19198782 |
|  |  | YFF96α (*rpl22*α::*RPL22***a**) x KN99**a** | 296390 | 18779002 |
|  |  | YFF113**a** (*rpl22***a**::*RPL22*α) x H99α (F) | 209531 | 18903233 |
|  |  | YFF96α (*rpl22*α::*RPL22***a**) x YFF113**a** (*rpl22***a**::*RPL22*α) | 627821 | 16875485 |
|  |  | YFF113**a** (*rpl22***a**::*RPL22*α) | 734591 | 18474185 |
|  |  | YFF96α (*rpl22*α::*RPL22***a**) | 1112111 | 17961966 |
|  |  | KN99**a** | 1114506 | 19499004 |
|  |  | H99α (F) | 1913287 | 20220771 |
| 3 | 2-129-3 | KN99**a** X H99α (F) | 1019883 | 19483790 |
|  |  | YFF96α (*rpl22*α::*RPL22***a**) x KN99**a** | 498288 | 17813433 |
|  |  | YFF113**a** (*rpl22***a**::*RPL22*α) x H99α (F) | 315538 | 19066478 |
|  |  | YFF96α (*rpl22*α::*RPL22***a**) x YFF113**a** (*rpl22***a**::*RPL22*α) | 1353861 | 18249658 |
|  |  | YFF113**a** (*rpl22***a**::*RPL22*α) | 841543 | 20359164 |
|  |  | YFF96α (*rpl22*α::*RPL22***a**) | 1094625 | 20118917 |
|  |  | KN99**a** | 1003989 | 21345286 |
|  |  | H99α (F) | 529033 | 19746327 |

**SUPPLEMENTAL FIGURE LEGENDS**


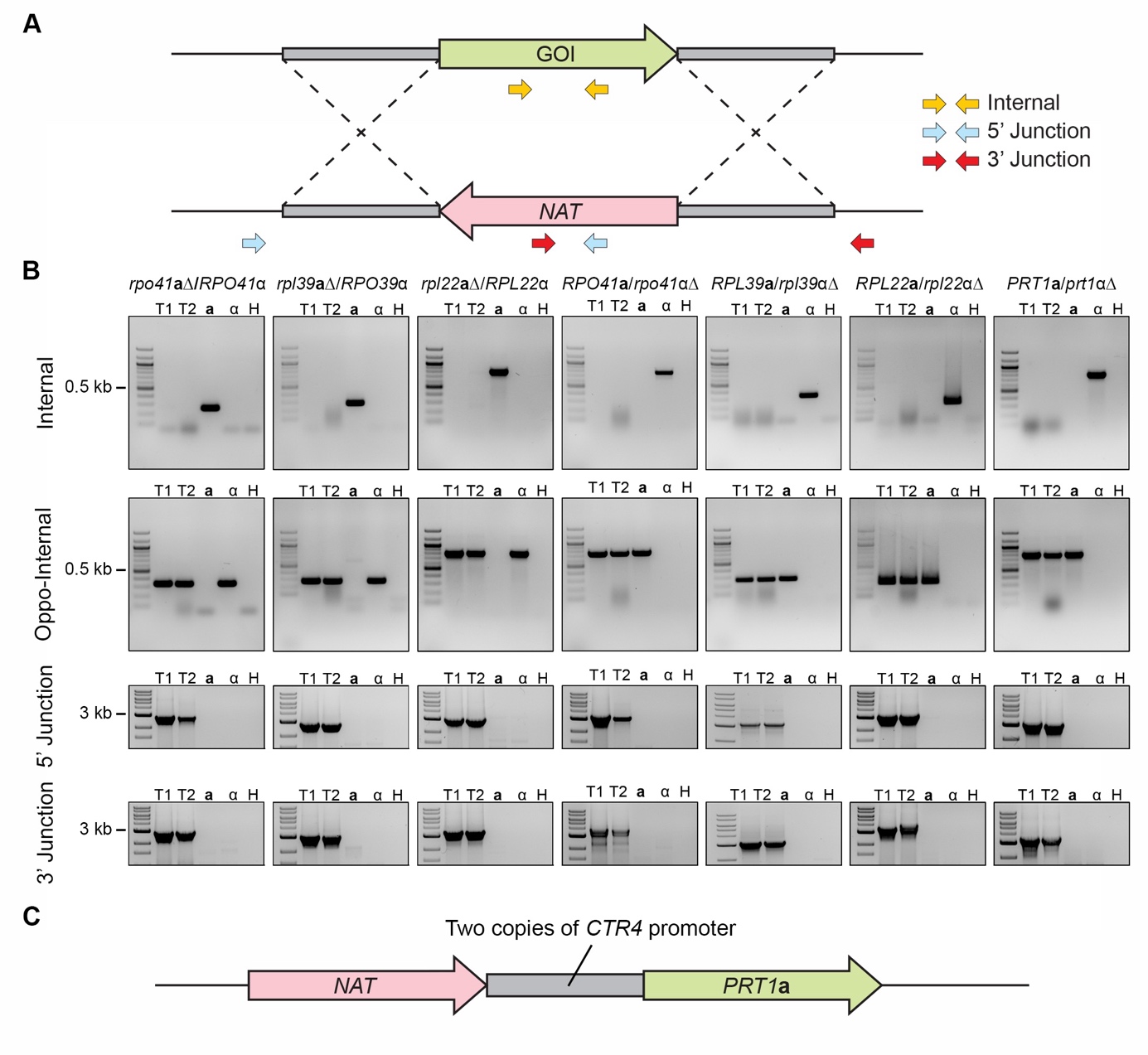


**FIG S1.** Genotypic validation of heterozygous deletion mutants. (A) Diagram of the gene replacement approach utilized to generate heterozygous deletion mutants and positions of primer pairs utilized for genotypic validation of transformants. (B) Genotyping of the heterozygous deletion mutants was conducted with primers targeting the internal regions of the ORFs of deleted alleles and alleles of the opposite mating type, as well as the 5’- and 3’-junctions of the deletion alleles; **a**, α, and H indicate the KN99**a**, KN99α, and water controls for PCR, respectively. See Fig. 2A and Fig. S5C for validation PCR of *myo2***a**Δ/*MYO2*α and *MYO2***a**/*myo2*αΔ, respectively. (C) Diagram of the tandem *CTR4* promoter insertion approach utilized to generate mutants for *PRT1***a**.


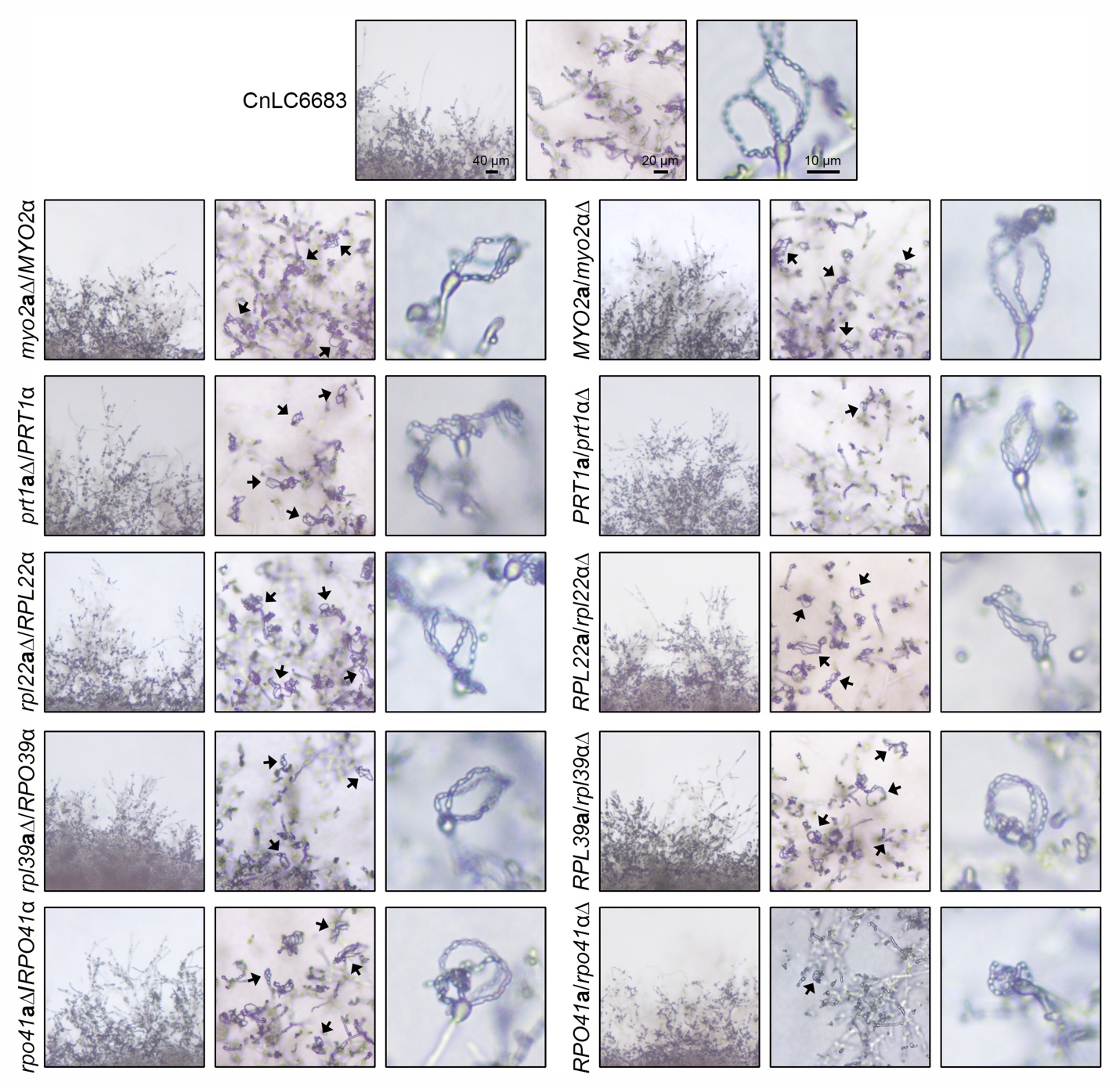


**FIG S2.** Selfing of heterozygous deletion mutants on MS media. Light microscopy images showing robust sporulation in all mutants except *PRT1***a**/*prt1*αΔ and *RPO41***a**/*rpo41*αΔ. Black arrows indicate spore chains. Selfing of *PRT1***a**/*prt1*αΔ and *RPO41***a**/*rpo41*αΔ exhibited robust hyphal development but infrequent sporulation events. Only one spore chain could be observed in the middle panel image of *PRT1***a**/*prt1*αΔ and *RPO41***a**/*rpo41*αΔ as indicated by black arrow. Scale bar is indicated in images of CnLC6683 samples. Scale bar = 40 μm (left), 20 μm (middle) and 10 μm (right)


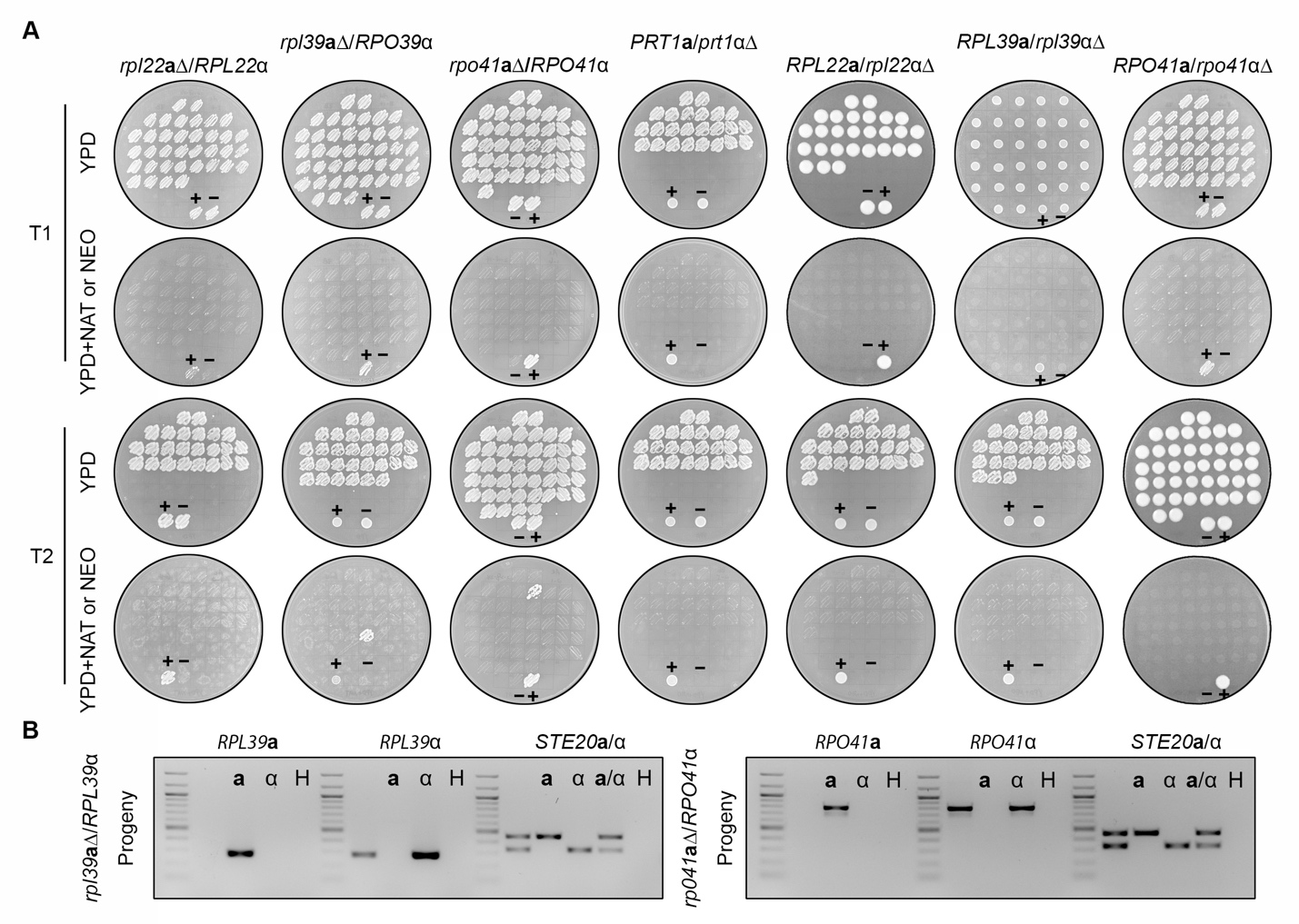


**FIG S3.** Phenotypic analyses of the random spores dissected from heterozygous deletion mutants. (A) Growth of the progeny on solid YPD and YPD supplemented with NAT (deletion of **a** alleles) or NEO (deletion of α alleles). The control (lower) patches are corresponding heterozygous mutants as positive control (+) and wild-type strain CnLC6683 as negative control (-). (B) The two drug resistant progeny from *rpl39***a**Δ/*RPL39*α and *rpo41***a**Δ/*RPO41*α were confirmed to still possess the wildtype allele of the opposite mating type, indicating these resistant progeny are aneuploid for the mating type locus chromosome.


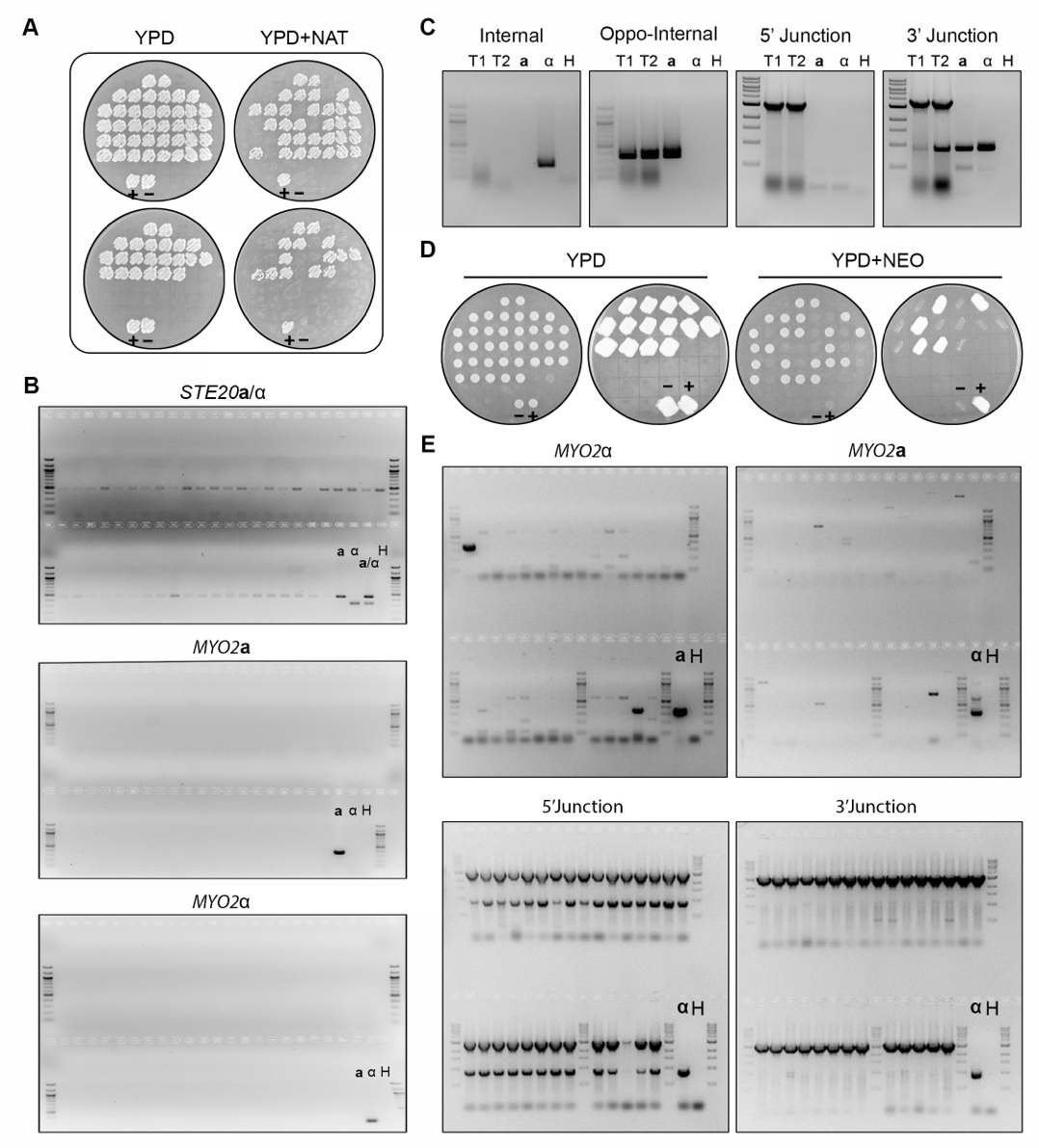


**FIG S4.** *MYO2***a** and *MYO2*α are not essential. (A) Phenotyping of randomly dissected spores of independent *myo2***a**Δ**/***MYO2*α mutant on YPD and YPD+NAT plates. The control (lower) patches are corresponding heterozygous mutants as positive control (+) and wild-type strain CnLC6683 as negative control (-). (B) Random spores dissected from *myo2***a**Δ**/***MYO2*α heterozygous mutants for mating type were genotyped to show the absence of both *MYO2***a** and *MYO2*α. (C) The *MYO2***a/***myo2*αΔ heterozygous mutants were genotypically validated. (D) Random spores dissected from the two independent *MYO2***a/***myo2*αΔ mutants were phenotyped on solid YPD and YPD+NEO plates. Bottom two patches are corresponding heterozygous mutants as positive control (+) and wild-type strain CnLC6683 as negative control (-). (E) Genotyping of *MYO2***a/***myo2*αΔ spores for the absence of both *MYO2***a** and *MYO2*α alleles is shown.


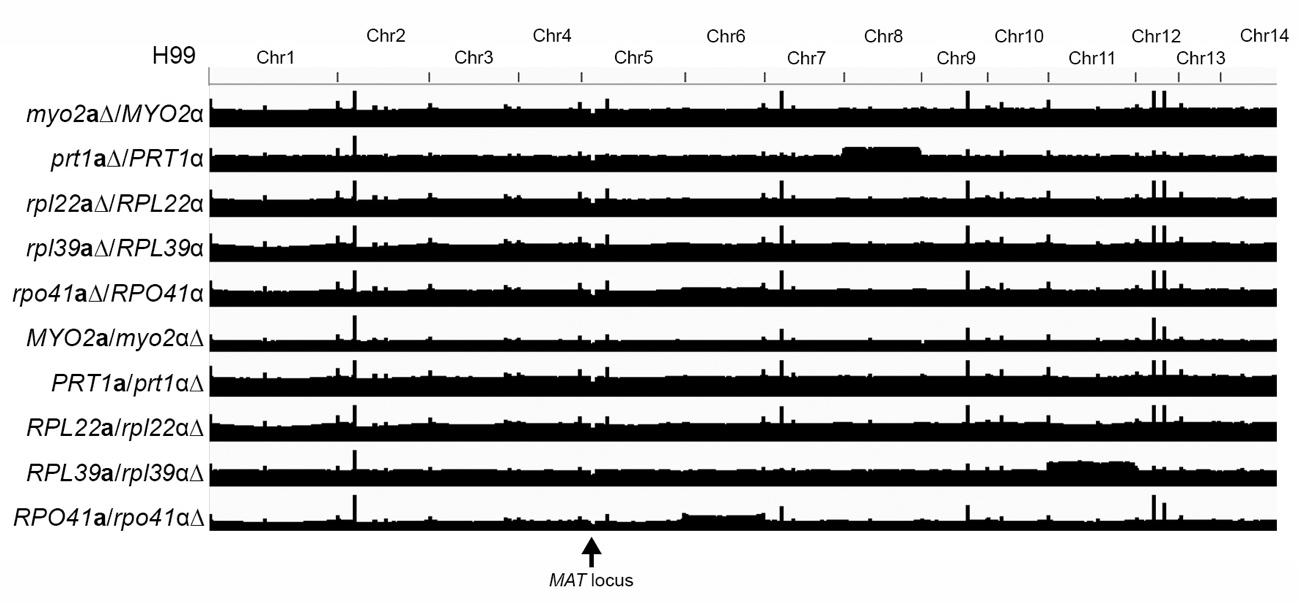


**FIG S5.** Genome-wide read depth analyses of heterozygous deletion mutants with Illumina whole genome sequencing reads. While mutant strains exhibited occasional aneuploidy for some chromosomes, there was no segmental deletion that was inside or linked to the mating type locus, confirming that the inviability observed in the meiotic progeny was due to the absence of the gene that was deleted.


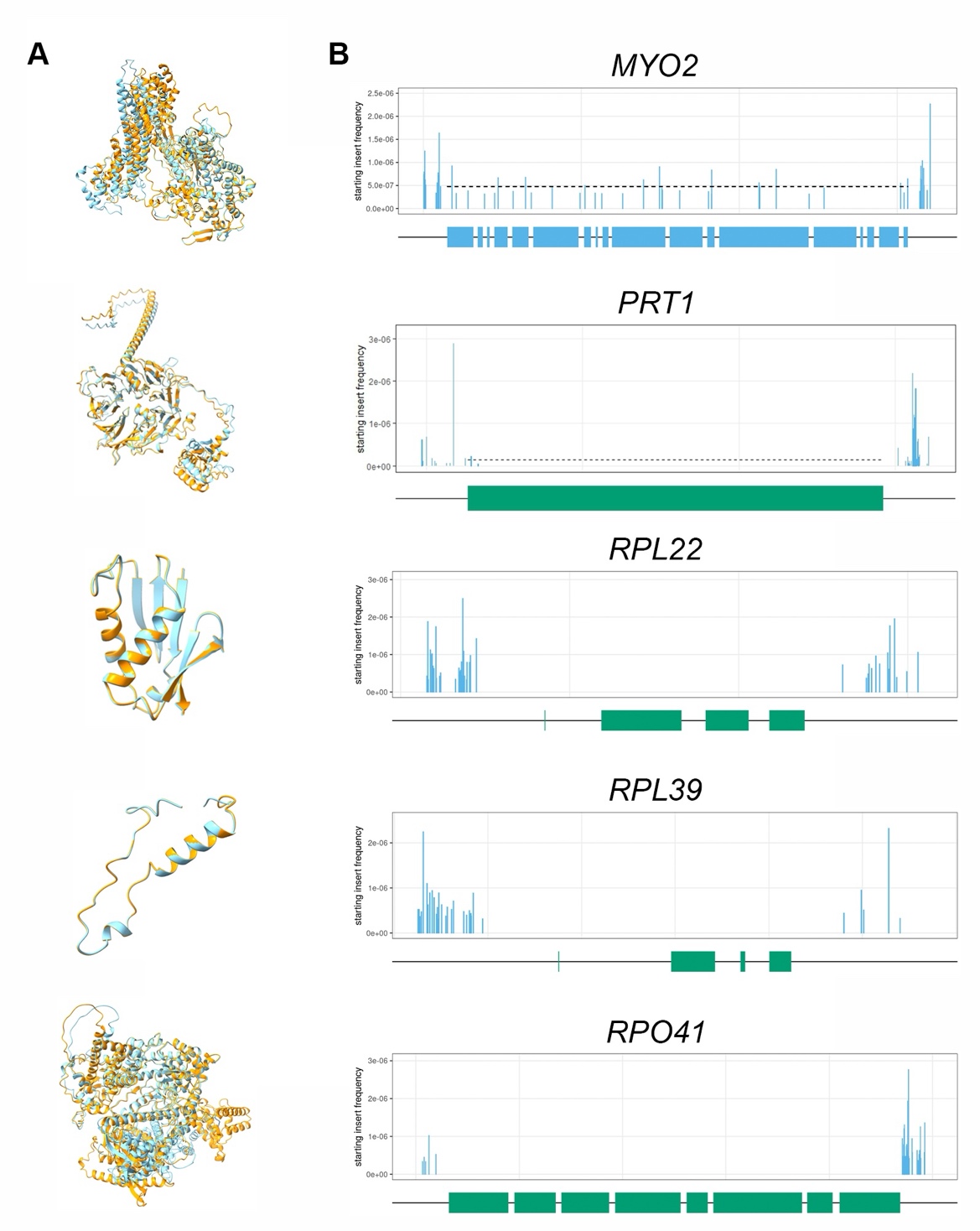


**FIG S6.** Predicted protein structures and essentiality for *MYO2*, *PRT1*, *RPL22*, *RPL39*, and *RPO41*. (A) Protein structures encoded by the **a** and α alleles for each gene were predicted using AlphaFold and aligned using the program ChimeraX, with **a** alleles depicted in orange and α alleles in blue. (B) Predicted essentiality of the α allele of each gene based on Tn-seq analyses (https://simrcompbio.shinyapps.io/Crypto_TN_seq_viewer/).

**REFERENCES**

1. Nielsen K, Cox GM, Wang P, Toffaletti DL, Perfect JR, Heitman J. 2003. Sexual cycle of *Cryptococcus neoformans* var. *grubii* and virulence of congenic a and alpha isolates. Infection and immunity 71:4831-4841 <http://dx.doi.org/doi:10.1128/IAI.71.9.4831-4841.2003>.

2. Peterson PP, Choi JT, Fu C, Cowen LE, Sun S, Bahn Y, Heitman J. 2024. The *Cryptococcus neoformans* STRIPAK complex controls genome stability, sexual development, and virulence. bioRxiv doi:10.1101/2024.08.06.606879 <http://dx.doi.org/doi:10.1101/2024.08.06.606879>.

3. So YS, Lee DG, Idnurm A, Ianiri G, Bahn YS. 2019. The TOR pathway plays pleiotropic roles in growth and stress responses of the fungal pathogen *Cryptococcus neoformans*. Genetics 212:1241-1258 <http://dx.doi.org/doi:10.1534/genetics.119.302191>.
